# Combining the tumor abnormal protein test with tests for carcinoembryonic antigens, cancer antigen 15–3, and/or cancer antigen 125 significantly increased their diagnostic sensitivity for breast cancer

**DOI:** 10.1097/MD.0000000000021231

**Published:** 2020-07-17

**Authors:** Rui Chen, Chaojun Jiang, Qiannan Zhu, Sainan You, Yan Li, Shuo Li, Lei Ding, Haojie Meng, Yuxin Yang, Xiaoming Zha, Jue Wang

**Affiliations:** a Department of Breast Disease, The First Affiliated Hospital of Nanjing Medical University; b Nanjing Medical University, Nanjing, China.

**Keywords:** breast cancer, cancer antigen 15-3, carcinoembryonic antigen, tumor abnormal protein

## Abstract

**Background:**

The tumor abnormal protein (TAP) test is used to screen for many cancers, but its use for breast cancer has not been studied.

**Methods:**

Tests for carcinoembryonic antigen (CEA), cancer antigen 125 (CA125), cancer antigen 15-3 (CA15-3), and TAP were administered to 261 women with operable benign breast disease and 348 with breast cancer. The cutoff value used for TAP was the mean + 3 standard deviations for benign breast disease patients (275.64 μm^2^). Sensitivities and specificities of single biomarker tests and combined tests were compared. The combined tests were defined as positive if any single biomarker was positive, and negative otherwise.

**Results:**

The single biomarker test sensitivities were similar: CEA, 7.18%; CA125, 4.89%; CA15-3, 7.47%; and TAP, 4.89%. For the combinations TAP + CEA + CA125, TAP + CEA + CA15-3, TAP + CA125 + CA15-3, and TAP + CEA + CA125 + CA15-3, the sensitivities were 16.67%, 17.82%, 16.38%, and 21.84%, respectively, and the specificities were 93.49%, 97.70%, 93.87%, and 92.72%.

**Conclusions:**

The 4-test combination showed the highest sensitivity (21.84%) and may be auxiliary used in early screening. TAP + CEA + CA15-3 showed high specificity (97.70%) and so could be used for confirming breast cancer.

## 1 Introduction

Breast cancer is one of the most common cancers in women.^[1]^ Generally, it is clinically diagnosed based on physical and radiological examinations. Serum tumor biomarkers for breast cancer, such as carcinoembryonic antigen (CEA), cancer antigen 125 (CA125), and cancer antigen 15-3 (CA15-3) have low sensitivity^[2]^ and so are typically used for follow-up monitoring rather than for early diagnosis.^[3,4]^ There is therefore a need for new serum tumor biomarkers or for the development of new diagnostic methods for breast tumor screening.

The glycoproteins produced by mutant genes have longer sugar chains and more complicated branching structures than those produced by wild-type genes, and these can be detected in peripheral blood.^[5]^ Tumor abnormal protein (TAP) is a collective term for the abnormal glycoproteins, calcium-histone complexes, and other substances commonly produced during the development of malignant tumors.^[5]^ TAP can be detected by a multistage coupling condensation reaction. First, coagulants bind to the various abnormal sugar chain glycoproteins to form primary condensates. Then, the primary condensates agglomerate to form secondary condensates bridged by calcium-histone. Finally, condensed particles form, and these can be detected and measured under a microscope. TAP testing has been reported to have diagnostic and/or prognostic value for gastric cancer^[6,7]^ bladder cancer,^[5]^ and colorectal cancer.^[8]^ However, its value for breast cancer diagnosis has not been studied.

The aim of this study was to investigate the diagnostic value of the serum TAP test for breast cancer, both alone and in various combinations with 3 traditional tumor biomarkers (CEA, CA125, and CA15-3).

## 2 Patients and methods

Between January 2017 and December 2018, 609 female patients who attended our breast disease department with an operable condition (benign or breast cancer) were enrolled in this study. The mean age was 47.2 ± 12.8 years (range, 12–92 years). This study was approved by the Ethics and Research Committee of our university hospital. Informed consent was obtained from all the patients in the study, and all the procedures were in accordance with the principles of the Declaration of Helsinki.

On admission, fasting blood samples (5 mL) were drawn from a peripheral vein and stored in K2 EDTA tubes (BD Vacutainer; Becton, Dickinson and Company, Franklin Lakes, NJ). The widely used tumor markers CEA, CA125, and CA15-3 were detected by electrochemiluminescence immunoassays (Roche Diagnostics GmbH, Basel, Switzerland). The cutoff values for CEA, CA125, and CA15-3 used to indicate a positive result for breast cancer were 4.7, 35.0, and 25.0 U/mL, respectively.

The TAP test was administered as follows. Blood (2 mL) was obtained from a peripheral vein and stored in a BD Vacutainer (Becton, Dickinson and Company). From this, 25 μL of blood was drawn to prepare each of the 2 blood smears needed for diagnosis. After the smears had dried naturally, 3 droplets (at about 50 μL/droplet) of agglutination agent (TAP detection kit; Zhejiang Ruisheng Medical Technology, Cixi, China) were added vertically onto them with a dropper. The smears were air dried for about 2 hours, and the agglomerated particles were then observed under a microscope and the area of condensed particles was measured by a TAP image specialist, who was an experienced professional pathologist. The cutoff value for the TAP test was calculated as the mean + 3 standard deviations for the TAP test results for the study patients with benign breast disease.

The results of CEA, CA125, CA15-3, and TAP tests for the patients with breast cancer were compared singly and in combinations, calculating the sensitivities and specificities of each single test and combination for the detection of breast cancer. The predictive value test (PV), positive predictive value (PPV) and negative predictive value (NPV) of the combination tests were also compared. For the combined tests, a result was defined as positive if 1 or more single biomarker was positive; otherwise, it was considered to be negative. Tumor size, nodal status, TNM stage, and molecular type were recorded after surgery.

### 2.1 Statistical analyses

The age of the patients at diagnosis and menopause were compared with 1-way ANOVA. The results of the TAP test were compared between the patient groups with benign breast disease and breast cancer by 2-tailed *t* test. The sensitivities, specificities, PV, PPV and NPV were compared with McNemar χ^2^ test.^[9]^ Stata (version 13) was used for the statistical analysis, and *P* < .05 was considered to indicate statistical significance.

## 3 Results

After core needle biopsy and/or surgery, 261 of the 609 patients were confirmed as having benign breast disease, and the remaining 348 patients were diagnosed with breast cancer. The mean ages of the benign breast disease and breast cancer patient groups were 40.8 ± 11.0 and 52.0 ± 12.0 years, respectively (*P* > .05). Data about the tumor size were available for only 287 of the patients with breast cancer, and data about nodal status were available for 288. Table 1 summarizes the tumor size, nodal status, and TNM stages of the patients with breast cancer.

**Table 1:**
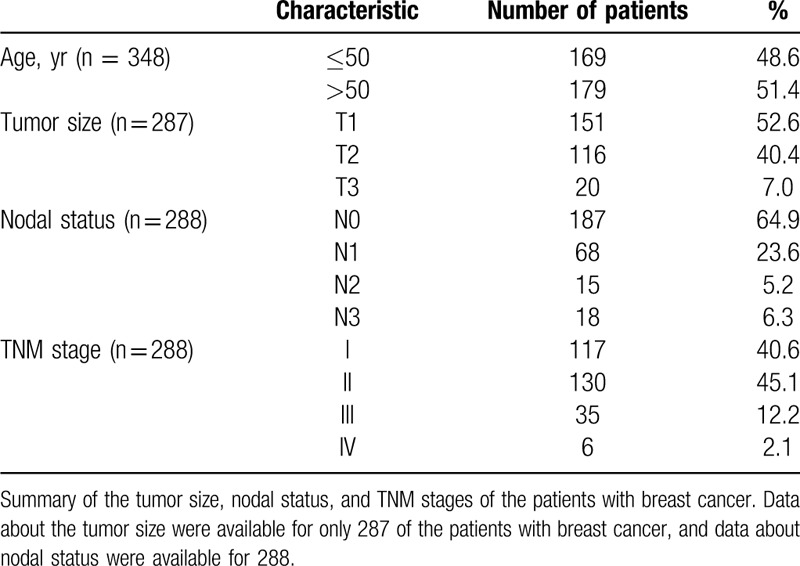
Age and tumor characteristics of the patients with breast cancer.

**Figure 1:**
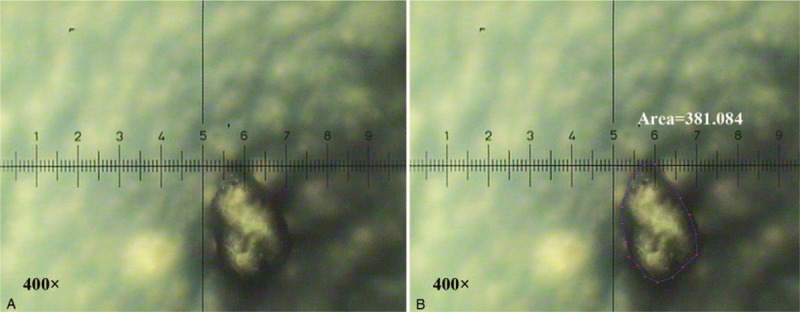
A positive tumor abnormal protein (TAP) test result, defined by the area of condensed particles exceeding the cutoff value of 275.64 μm^2^. (A) Detection of TAP. (B) Measurement of the area of TAP. Original magnification 400 × . TAP = tumor abnormal protein.

In the TAP test, the mean area of condensed particles was 134 ± 47 μm^2^ in the benign breast disease group and 161.75 ± 65.26 μm^2^ in the patients with breast cancer. Based on the mean TAP results for the patient group with benign breast disease, the cutoff value indicating a positive result for breast cancer was set as 275.64 μm^2^ (mean + 3 standard deviations). As a result, 1 of the patients with benign breast disease and 17 of the breast cancer patients were classified as TAP test positive (Fig. 1). Thus, the sensitivity and specificity of the TAP test for diagnosing breast cancer were 4.89% and 99.62%.

**Figure 2:**
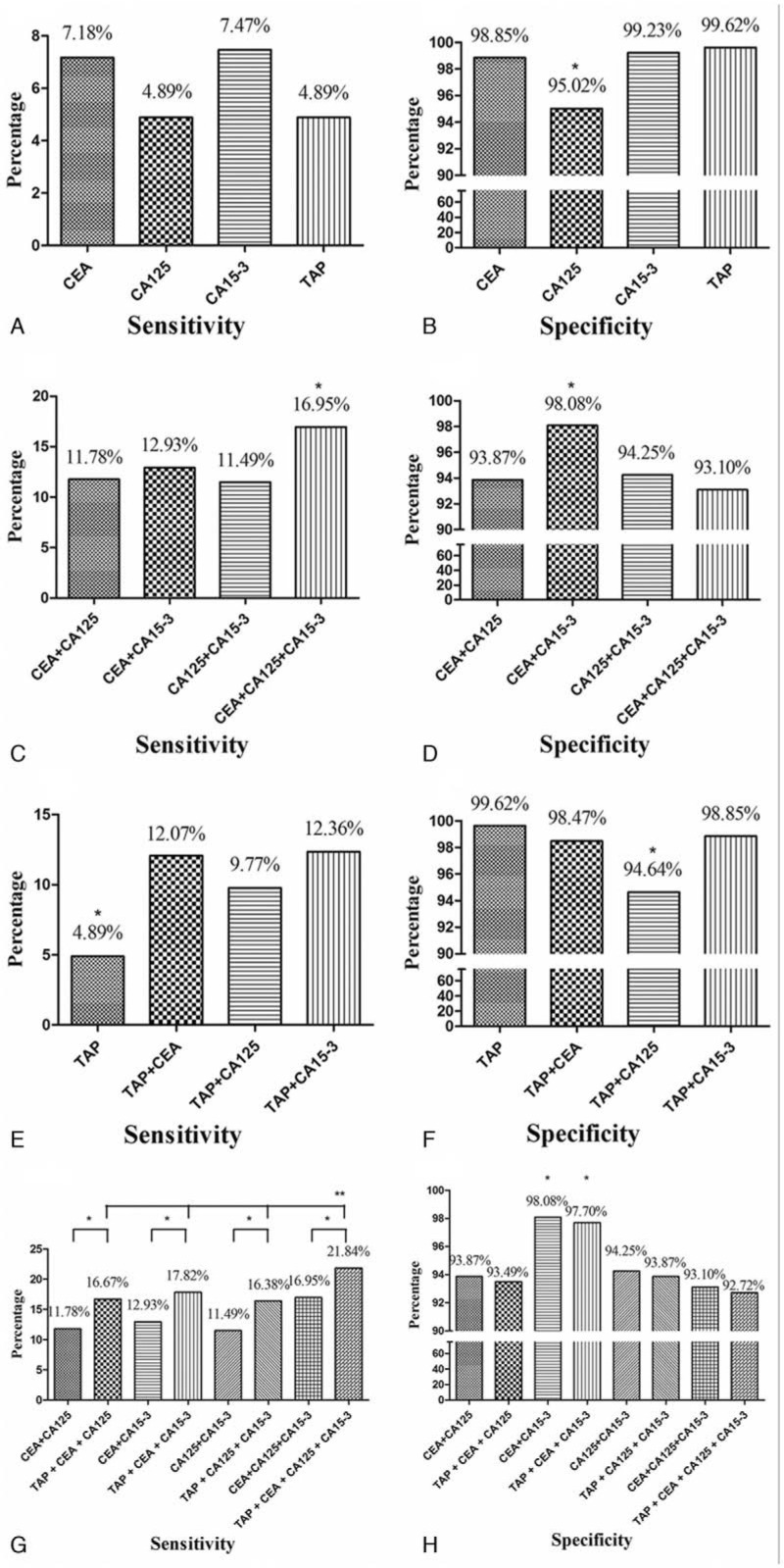
Sensitivities and specificities of the single biomarker tests and combinations of tests. The sensitivities of the single biomarker tests (carcinoembryonic antigen (CEA), cancer antigen 125 (CA125), cancer antigen 15-3 (CA15-3), and tumor abnormal protein (TAP)) were similar (A), although the specificity of the CA125 test was lower than those of the other 3 tests (B). Among the combination of tests without the TAP test (CEA + CA125, CEA + CA15-3, CA125 + CA15-3, and CEA + CA125 + CA15-3), the highest sensitivity was shown by the combination CEA + CA125 + CA15-3 (C), and the highest specificity was shown by CEA + CA15-3 (D). When the TAP test was combined with the CEA, CA125, and CA15-3 tests separately, all the sensitivities increased (E), and only the specificity of TAP + CA125 showed a decrease (F). When the TAP test was added to the various combinations of CEA, CA125, and CA15-3 tests, the sensitivities of the combinations all increased, with all 4 tests together (TAP + CEA + CA125 + CA15-3) showing the highest sensitivity (G). The specificities did not decrease with the addition of the TAP test. The combinations CEA + CA15-3 and TAP + CEA + CA15-3 showed the highest specificities (H). ^∗^*P* < .05; ^∗∗^*P* < .05. CA125 = cancer antigen 125, CA15-3 = cancer antigen 15-3, CEA = carcinoembryonic antigen, TAP = tumor abnormal protein.

Figure 2 presents the sensitivities and specificities of the single and combined tests. The numbers of patients with breast cancer who tested positive for CEA, CA125, CA15-3, and TAP were 25, 17, 26, and 17, respectively. The test with the lowest specificity was for CA125 (95.02%), compared with CEA (98.85%), CA15-3 (99.23%), and TAP (99.62%) (*P* < .05; Fig. 2B).

The sensitivities for CEA + CA125, CEA + CA15-3, and CA125 + CA15-3 were 11.78%, 12.93%, and 11.49%, respectively. In comparison, the sensitivity for the combination CEA + CA125 + CA15-3 (16.95%) was significantly higher (*P* < .05; Fig. 2C). The combination CEA + CA15-3 had the highest specificity (98.08%) among the combined tests (*P* < .05; Fig. 2D).

Combining the TAP test with each of the CEA, CA125, or CA15-3 tests separately resulted in significant increases in sensitivity (*P* < .05; Fig. 2E). The sensitivities of TAP + CEA, TAP + CA125, and TAP + CA15-3 were 12.07%, 9.77%, and 12.36%, separately. The specificities of TAP + CEA (98.47%) and TAP + CA15-3 (98.85%) were similar to that of TAP (99.62%); however, the specificity of TAP + CA125 was lower (94.64%) (*P* < .05; Fig. 2F).

The TAP test was added to the combinations CEA + CA125, CEA + CA15-3, CA125 + CA15-3, and CEA + CA125 + CA15-3, resulting in an increase in their sensitivities, to 16.67%, 17.82%, 16.38%, and 21.84%, respectively (*P* < .05; Fig. 2G). The combination of all 4 tests (TAP + CEA + CA125 + CA15-3) showed the highest sensitivity (*P* < .05; Fig. 2G). Adding the TAP test to the combinations CEA + CA125, CEA + CA15-3, CA125 + CA15-3, and CEA + CA125 + CA15-3 did not result in any substantial decreases in the specificities (93.49%, 97.70%, 93.87%, and 92.72%, respectively) (*P* < .05, Fig. 2H). Among these combinations, the 2 with the highest specificities were CEA + CA15-3 and TAP + CEA + CA15-3, at 98.08% and 97.70%, respectively (*P* < .05; Fig. 2H).

**Figure 3:**
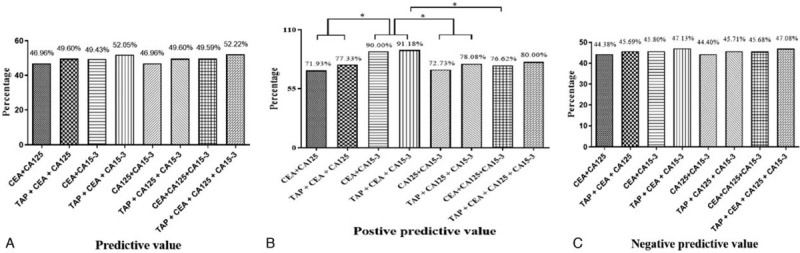
The predictive value, positive predictive value, and negative predictive value of different combined tests. Predictive values for all combinations with or without tumor abnormal protein (TAP) test were similar (A). Combinations of carcinoembryonic antigen + cancer antigen 15-3 and TAP + carcinoembryonic antigen + cancer antigen 15-3 had higher positive predictive value than may other combinations (B). Negative predictive value for all combinations with or without TAP test were similar (A). ^∗^*P* < .05; ^∗∗^*P* < .05. TAP = tumor abnormal protein.

The PVs for CEA + CA125, CEA + CA15-3, CA125 + CA15-3 and CEA + CA125 + CA15-3 were around 48%. No obvious improvement occurred even TAP test was added into above combinations (Fig. 3A). Combinations of CEA + CA15-3 and TAP + CEA + CA153 had higher PPV than many other combinations (Fig. 3B). Finally, all combinations had similar NPV (Fig. 3C).

## 4 Discussion

CEA, CA125, and CA15-3 are glycoproteins whose glycan profiles change with cancer progression. CEA was the first serum tumor biomarker to be identified, in 1965; it is overexpressed in, for example, most colon cancer patients and some breast cancer patients.^[10]^ CA125 is a biomarker for ovarian cancer, and it has been shown to be upregulated in breast cancer tissues.^[11]^ CA15-3 is a transmembrane glycoprotein that is overexpressed in carcinomas of epithelial origin, including breast, ovarian, and pancreatic cancers. It is a powerful prognostic indicator for advanced breast cancer, as well as an independent predictor of breast cancer recurrence.^[12,13]^ The tests for serum CEA, CA125, and CA15-3 are routinely used for patients with breast cancer^[14]^; however, their low sensitivities limit their clinical value. In the present study, the sensitivities of the CEA, CA125, and CA15-3 tests applied separately were as low as 4.89% to 7.47%.

To increase the sensitivity of the screening, all the various combinations tests of the 3 biomarkers were compared. For this, a combined test result was defined as positive if any single biomarker was positive, and negative otherwise. First, CEA, CA125, and CA15-3 were combined and analyzed without the TAP test. The combined test CEA + CA125 + CA15-3 had the highest sensitivity (16.95%). Because the CA125 test alone had a lower specificity than those of the CEA and CA153 tests, the combined test CEA + CA15-3, which did not contain CA125, had a higher specificity (98.08%) than the combined tests containing CA125.

The use of liquid biopsies for tumor diagnosis is becoming increasingly popular, and novel tumor biomarkers, such as cell-free DNA and circulating tumor cells, ^[15]^ have been discovered and developed. These biomarkers are expressed in different biological tissues and can reveal the presence of malignancies. TAP is a complex of abnormal glycoproteins, calcium-histone, and various substances expressed by genes when normal cells become cancerous, regulating the cell cycle of tumor cells. When the level of TAP increases sufficiently, it can be detected in peripheral blood.^[16]^ Zhang et al have reported positive rates for TAP tests of 78.16% in patients with bladder cancer and 10.81% in patients without tumors (*P* < .01).^[5]^ In gastric cancer, the positive rate for TAP tests was 64.3%, and this was an independent negative predictive factor for progression-free survival.^[17]^ The TAP test has also been shown to be sensitive for monitoring the responsiveness to palliative chemotherapy in patients with advanced gastric cancer.^[18]^ As with colorectal cancer, studies have shown that the TAP test had high sensitivity and specificity for gastric cancer and that it can be used as a new independent indicator for monitoring the effect of chemotherapy.^[8]^ However, as yet, there have been no data available for its use in breast cancer.

In the present study, the mean area of condensed particles in the TAP tests administered to the patients with benign breast diseases was 134.49 ± 47.05 μm^2^; the cutoff value for the TAP test was therefore set as 275.64 μm^2^ (the mean + 3 standard deviations). When this threshold was applied across the TAP test results for all the patients, the sensitivity and specificity of the test for detecting breast cancer were 4.89% and 99.62%, respectively. Although the TAP test did not have superior sensitivity to any of the other 3 biomarker tests, it had a higher specificity than CA125.

The TAP test involves a different detection mechanism from that of the traditional glycoprotein tests. As a result, most of the TAP-positive patients were not positive for CEA, CA125, or CA15. We therefore combined the TAP test with each of the CEA, CA125, and CA15-3 tests separately. Doing so increased the sensitivities of all 3 tests, and the only specificity to decrease was that for the combination with CA125. The TAP test was then added to the various combinations of 2 or 3 of the traditional biomarker tests. This further increased the sensitivities of those combined tests. The combination with the highest sensitivity was all 4 tests together (TAP + CEA + CA125 + CA15-3), with a sensitivity of 21.84%. However, because the CA125 test had the lowest specificity for breast cancer diagnosis, the specificity for the combination of all 4 tests together was lower than for the combinations without the CA125 test. In particular, the CEA + CA15-3 and TAP + CEA + CA15-3 combinations, which did not include the CA125 test, had the highest specificities. In addition, the PPV of TAP + CEA + CA15-3 test was also higher than the test of CA125 + CEA + CA15-3.

A limitation of this preliminary study is that we enrolled only 261 benign breast disease patients, which may have influenced the accuracy of the TAP cutoff value. In addition, only 17 of the 348 breast cancer patients tested positive for TAP, and we were unable to stratify these patients on the basis of tumor size, nodal status, TNM stage, or molecular subtype. Further studies with a larger sample size and stratification are needed to confirm the clinical significance of TAP test for breast cancer diagnosis. In addition, the combination of clinical, imaging, and pathological findings is exceptionally reliable and sensitive to detect early breast cancer. The addition of these ancillary tests with low sensitivities offered limited value in early detection of breast cancer. However, as a simple and convenient detection method, it may be used as a screening and review indicator for breast cancer, we look forward to further researches.

## 5 Conclusions

The TAP test involves a different detection mechanism from those of the traditional serum biomarkers CEA, CA125, and CA15-3. The combination of all 4 tests, TAP + CEA + CA125 + CA15-3, showed the highest sensitivity for the diagnosis of breast cancer (21.84%); this combination might therefore be auxiliary in the early screening of breast cancer. Conversely, the combination TAP + CEA + CA15-3 had a very high specificity (97.70%) and PPV (91.18%), and it might be helpful for confirming breast cancer.

## Author contributions

X Zha and J Wang designed and supervised the study; R Chen, C Jiang collected the data of patients; Q Zhu and S You confirmed the data; R Chen and C Jiang did the statistical analysis; Y Li, S Li, L Ding, H Meng, Y Yang wrote the manuscript. All authors have revised the final version.
